# Genetic Sequence Variants in *TLR4*, *MBL* or *IL-1* Receptor Antagonist is not Associated to Increased Risk for Febrile Neutropenia in Children with ALL

**DOI:** 10.3390/children7120296

**Published:** 2020-12-16

**Authors:** Martina Wahlund, Malin Lindqvist Appell, Ida Hed Myrberg, Anna Berggren, Anna Nilsson

**Affiliations:** 1 Department of Medicine Solna, Division of Infectious Diseases, Karolinska Institutet, 171 64 Stockholm, Sweden; martina.wahlund@ki.se (M.W.); anna.berggren@ki.se (A.B.); 2 Department of Clinical Microbiology, Karolinska University Hospital, 171 64 Stockholm, Sweden; 3 Department of Medical and Health Sciences, Division of Drug Research, Linkoping University, 581 83 Linkoping, Sweden; malin.lindqvist.appell@liu.se; 4 Childhood Cancer Research Unit, Department of Women’s and Children’s Health, Karolinska Institutet, 171 65 Stockholm, Sweden; ida.hed.myrberg@ki.se; 5 Astrid Lindgren Children’s Hospital, Karolinska University Hospital, 171 64 Stockholm, Sweden

**Keywords:** febrile neutropenia, acute lymphoblastic leukemia, *TLR4*, *MBL*, *IL-1Ra*

## Abstract

Sequence variants in genes involved in the immune system have previously been linked to neutropenia as well as infections in cancer patients. Sequence variants in genes coding for *TLR4*, *MBL*, and *IL-1Ra* were investigated in relation to clinical utility of identifying severe episodes of febrile neutropenia (FN) in a cohort of children undergoing treatment for acute lymphoblastic leukemia. The study included 122 children, where data on FN and microbiological findings were retrospectively collected from medical records. Sequence variants in genes coding for *MBL*, *TLR4*, and *IL-1Ra* were identified by pyrosequencing, TaqMan SNP genotyping assay, and gel electrophoresis. A total of 380 episodes of FN were identified and in 139 episodes, there was a microbiological defined infection. Age and treatment intensity were all associated with the risk of developing FN. No sequence variant was associated to increased numbers of FN episodes. Two sequence variants in the *TLR4* gene increased the risk of viral infection, whilst sequence variants in the *IL-1Ra* gene were associated to a decreased risk of bacterial blood-stream infection (BSI). The investigated sequence variants did not associate with increased risk for FN or to severe infections, as to why the clinical utility as a risk-stratification tool is low. Most episodes of FN were classified as fever with unknown origin, emphasizing the need for improved microbial detection methods.

## 1. Introduction

Acute lymphoblastic leukemia (ALL) is the most frequent cancer in children. The long-term survival of children with ALL has increased considerably [1], although complications associated with chemotherapy are common. Still, infections remain as a cause of increased morbidity and mortality [2,3]. Data on febrile neutropenia (FN) and microbiological defined infections (MDI) during the entire pediatric ALL treatment period is limited. Recent studies have reported that bacterial bloodstream infections (BSI) and invasive fungal disease (IFD) still account for most infection-related deaths. Viral infections, mainly respiratory infections, are also commonly detected in FN [3,4,5,6]. Thus, early identification of children at risk of severe infections during chemotherapy could be a way forward and lead to improved antibiotic treatment strategies for a subgroup of children with ALL.

The great variability in the frequency and severity of infections in children with ALL implies an individualized susceptibility to infection. Previous studies have associated sequence variants in genes coding for the innate immune proteins Toll-like receptor 4 (*TLR4*), mannose-binding lectin (*MBL*), and interleukin-1 receptor antagonist (*IL-1Ra*) to increased risk for either bacterial or viral infection during cancer therapy [7,8,9,10,11,12]. Sequence variants in the *TLR4* have been associated with an increased risk of sepsis, Gram-negative BSI, as well as the development of chemotherapy-induced neutropenia [11,12,13]. Data regarding sequence variants in the *MBL* and risk of FN in children during chemotherapy are inconclusive, with studies indicating an increased risk of bacterial infection, whilst some studies do not suggest any correlation to FN or any other MDI [9,10,11,14,15]. The IL1RN*2 allele has been associated with an increase in the susceptibility to sepsis and clinically severe sepsis in adults, but also to an increased risk of septic shock in children with ALL [7,16,17].

Previous studies lack stringency in patient cohorts where data for children with different cancers are compared as well as where patients on different treatment protocols are compared for these gene sequence variants. In addition, definitions of etiology of FN have been lacking. Therefore, the primary aim of the current study was to assess the clinical utility of these gene sequence variants in risk stratification of FN in a homogenous cohort of pediatric ALL patients.

## 2. Materials and Methods

### 2.1. Study Cohort

Children diagnosed with ALL between May 2004 and April 2014 at Astrid Lindgren Children’s Hospital (Stockholm, Sweden), and subsequently treated within the Nordic Society for Pediatric Hematology and Oncology (NOPHO) ALL 2000 and 2008 protocols, were retrospectively included in the study. The NOPHO ALL registry was used for patient identification. The following exclusion criteria were used: (i) Resistant disease leading to change of treatment protocol, (ii) previous chemotherapy treatment, (iii) infant-ALL, (iv) Philadelphia chromosome-positive ALL, (v) germ-line chromosomal abnormalities, (vi) known immunodeficiencies, (vii) missing sample, or (viii) insufficient treatment information in the medical records. The study was conducted in accordance with the Declaration of Helsinki, and the protocol was approved by the the Regional Ethical Review Board in Stockholm, Sweden (Dnr: 2016/442-31/2).

There were some differences in treatment intensity between the 2000 and 2008 protocol; children treated within the standard (SR) and intermediate risk (IR) groups in the 2008 protocol received slightly higher doses of chemotherapy. In the 2008 protocol, patients in the IR group received an extra delayed consolidation before the start of maintenance II. In addition, the subsequent maintenance II was supplemented with intrathecal methotrexate (MTX) at 8-week intervals. However, the main difference was that the high-risk (HR) groups received significantly more intensive chemotherapy with at least six high-dose chemotherapy blocks in the 2008 protocol as compared to the 2000 protocol.

### 2.2. Data Collection

Children were followed from the diagnosis of ALL to the end of treatment (2.5 years), until hematopoietic stem-cell transplantation (HSCT), or to relapse within the primary treatment protocol. FN was defined as two or more spikes at least one hour apart of a temperature ≥ 38.0 °C or a single spike ≥ 38.5 °C, with a neutrophil count of ≤1 × 10^9^ cells/L at the time of the fever or decreasing to ≤0.5 × 10^9^ cells/L within 48 h of fever onset [18]. Patient characteristics, numbers of FN episodes, microbiological findings (bacterial BSI, IFD, or viral infections) during the episodes of FN were retrospectively collected from patient charts.

Due to the retrospective approach of the study, all microbiological findings were controlled by two clinicians (AN, MW) using predefined criteria to decide whether the microbiological findings were relevant or not. Bacterial BSI with a positive blood culture were included if there was an intention-to-treat at the time of infection. Pathogens defined as contaminants by the laboratory and the clinician at the time of infection were excluded. For respiratory viral infections, the samples were analyzed using either viral isolation or immunofluorescence (until 30 September 2007) and thereafter, PCR on nasopharyngeal aspirates (NPA) [19]. Positive NPA samples were included only if they occurred simultaneously with respiratory symptoms. For gastrointestinal viral infections, children with repeated positivity were excluded due to the prolonged viral shedding of these viruses [20,21]. IFD was included only if there was a positive microbial finding in an otherwise sterile compartment corresponding to the European Organization for Research and Treatment of Cancer/Mycoses Study Group (EORTC/MSG) criteria of proven IFD [22].

### 2.3. Gene Sequence Variant Analysis 

In short, targeted sequences and the *IL1RN* sequence repeat were amplified in a total volume of 10 µL using 1 ng DNA, 1 × QIAGEN HotStarTaq Master Mix, with 2 mM MgCl_2_ and 0.4 µM individual PCR-primers and primers originally from Tarlow et al. for *IL1RN* (supporting information; Tables S1 and S2) [23]. The following PCR amplification conditions were used: 15 min at 95 °C; 45 cycles of 15 s at 95 °C, 90 s at 55 °C, and 30 s at 72 °C; 10 min at 72 °C.

Genotyping was performed using pyrosequencing (supporting information; Tables S1 and S2) to test for *MBL* rs1800450, rs5030737, rs1800451 and *TLR4* rs2737190, rs1927911, rs10759931, and rs11536889 as according to the manufacturer’s instructions as earlier described in Wahlund et al., 2019 [24]. For two sequence variants in *TLR4*, genotyping was performed using TaqMan™ (Applied Biosystems, Foster City, CA, USA) SNP Genotyping Assay detecting rs4986790 (assay IDC11722238) and rs4986791 (assay IDC11722237). In short, 5 ng DNA was mixed with 2 × TaqMan™ Genotyping Master Mix Cat#4371355, 0.25 µl of 20 × genotyping assay, in a total volume of 10 µl. The thermocycling and data analysis were performed with an ABI 7500 Real-Time PCR instrument (Applied Biosystems, Foster City, CA, USA) using standard mode. For *IL1RN*, the resulting product after PCR was detected by agarose gel electrophoresis (E Gel™ General Purpose Agarose Gels, 2%, Invitrogen, Carlsbad, CA, USA) to determine the size(s) of amplicon(s) corresponding to a variable number of repeats. A fragment of 410 bp corresponded to four repeats of the 86 bp sequence (IL1RN*1), 240 bp fragment to two repeats (IL1RN*2), and 500 bp to five repeats (IL1RN*3) [23].

### 2.4. Statistical Analysis

Descriptive statistics are presented as medians, inter-quartile range, and range for continuous variables, and frequencies and percentages for categorical variables. The association between covariates and time to FN and the different MDI, respectively, was evaluated using Andersen–Gill models, i.e., semi-parametric survival models allowing for recurring events, taking into account that one child may have had multiple episodes [25]. The function coxph in the R package survival was used to fit the models [26,27]. Episodes where more than one type of infection was detected (e.g., co-infection with bacteria and virus), were excluded from the final analysis. Censoring was made for end of treatment (typically 130 weeks after diagnosis), relapse, death, or HSCT.

First, the association between time to FN/MDI and the background variables age at diagnosis (years), phenotype (T-cell/pre-B ALL), risk group (SR/IR/HR), and protocol (NOPHO-ALL 2000/NOPHO-ALL 2008) were evaluated in univariable Andersen–Gill models. The linearity assumption of the age covariate was tested by comparing a model with a restricted 3-knot cubic spline basis for age to a model with a linear assumption for age, using a Wald test. For FN, bacterial BSI, and Gram-negative BSI, the linearity assumption did not hold, and age was adjusted for using restricted cubic splines. The association between sequence variants and time to FN/MDI was then evaluated in univariable models, and adjusted for statistically significant background variables in multivariable models. Adjustments for age, risk group, and protocol were made for FN, bacterial BSI, and Gram-negative BSI. Gram-positive BSI was adjusted for age and risk group and viral infections were adjusted for age and protocol. The proportional hazards assumption was checked by testing the correlation between the scaled Schoenfeld residuals and rank-ordered event times. R version 3.6.0 was used for all analyses [28].

## 3. Results

### 3.1. Patient Characteristics and Episodes of FN

Of the 157 identified children, 122 met the inclusion criteria (Table 1). The median age was 5.4 years (range 1.1–17.9), and 54, 48, and 20 children were treated within the SR, IR, and HR groups, respectively. Eight patients suffered from relapse within the treatment period (median time to relapse 80.9 weeks, range 35.9–120.6 weeks), two were transferred to HSCT, and two died within the follow up due to infectious-related complications.

**Figure 1:**
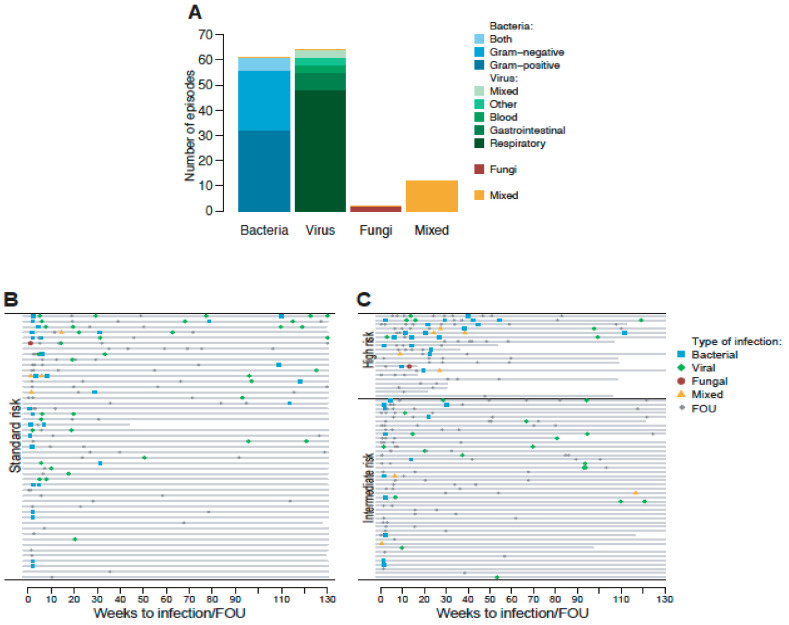
FOU and MDI during episodes of FN during the entire ALL treatment. (**A**) MDI (*n* = 139) within the 380 episodes of FN. Nine of the bacterial BSI and viral infections were co-infections with both virus and bacteria, six of the bacterial BSI were both Gram-positive and Gram-negative, and for the IFD, four were mixed infections where one combined with virus, two combined with bacterial BSI, and one combined with both virus and bacterial BSI. (**B**,**C**) Occurrence of FN episodes during the entire ALL treatment sorted after respective MDI or fever of unknown origin (FUO) and treatment intensity standard (SR), intermediate (IR) and high risk (HR). Each line represents one patient. The HR groups in the 2000 protocol were only treated for 104 weeks.

A total of 380 FN episodes were documented within the cohort of 122 children corresponding to a median of three episodes (range 0–11, interquartile range IQR 1–4) in each child. From the clinical diagnostic procedures, viral infection was identified in 64 (16.8%) of the FN episodes (median 0, range 0–5 episodes), bacterial BSI in 61 (16.1%) episodes (median 0, range 0–5 episodes), only IFD in two (0.5%) episodes, and twelve (3.2%) episodes were mixed infections (Figure 1A). In the majority of FN episodes (*n* = 240), no microbiologic pathogen was identified and these episodes were classified as fever of unknown origin (FOU). Bacterial BSI was more commonly detected during the first months of ALL treatment, but in HR-ALL also during the first year when intense therapy is administered. On the other hand, viral infections were present during the entire treatment period, especially in the SR- and IR-groups (Figure 1B,C).

**Figure 2:**
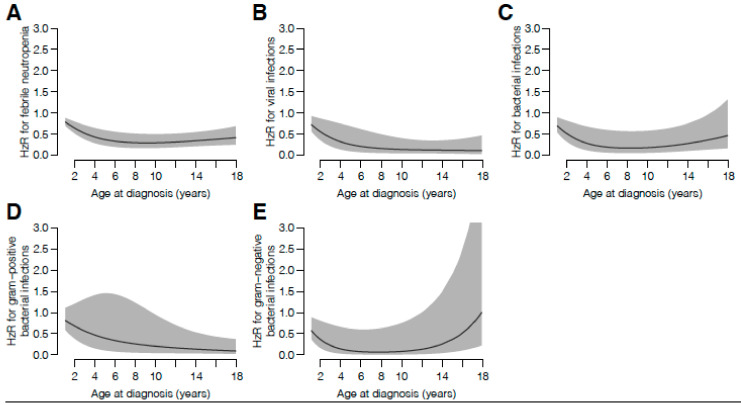
(**A**–**E**): Age impact on the risk of FN and respective MDI. Hazard ratio (HzR) for a one-year increase in age at diagnosis, calculated using 3-knot restricted cubic splines for age in Andersen–Gill models, for (**A**) febrile neutropenia, (**B**) viral infections, (**C**) bacterial BSI, (**D**) Gram-positive BSI, (**E**) Gram-negative BSI.

Thereafter, the overall risk of FN, as well as the risk for the respective MDI, were analyzed. Age at diagnosis was an identified risk factor of developing FN and the respective MDI, however, the association between age and the natural logarithm of the hazard function was not linear (Figure 2A). Younger age was more associated to an increased risk for viral infections and Gram-positive BSI, whilst Gram-negative BSI was associated to an increased risk in both the younger and older ages (Figure 2B–E). In addition, treatment intensity (the NOPHO ALL 2008 treatment protocol as well as treatment for the HR-groups) were associated with more episodes of FN.

### 3.2. Genetic Sequence Variants and the Overall Risk of FN and MDI

Thereafter, we performed genotyping, which was successful for the vast majority of the samples (except for two samples, one for the Rs4986790 and one for *IL1RN*). At least one of the different sequence variants investigated were identified in 117 (*TLR4*), 39 (*MBL2*), and 55 (*IL1RN*) children (supporting information, Tables S3 and S4), thus some children harbored more than one sequence variant, both within the same gene and in between the genes investigated.

The analysis of microbiological findings in relation to sequence variants was stringently performed where only bacterial BSI episodes (*n* = 61, viral and IFD excluded) or only viral episodes (*n* = 64, bacterial and IFD excluded) were taken into account, respectively. Thereafter, a sub-analysis of episodes with Gram-positive or Gram-negative bacteria was performed (co-presence of Gram-positive and Gram-negative bacteria were excluded).

When comparing the different sequence variants to all the episodes of FN (Table 2), as well as to the respective MDIs, two sequence variants in *TLR4*; rs10759931 and rs11536889 were associated with an increased risk of viral infections. For sequence variants in the *IL1 Ra* gene, children with at least one sequence variant (heterozygous or homozygous) in both the IL1RN*2 allele and IL1RN*3 allele were grouped together and thereafter identified to have a decreased risk of bacterial BSI as compared to wild-type children. In addition, further analysis of the bacterial infections (either Gram-positive (*n* = 32) or Gram-negative BSI (*n* = 24)) showed that the IL1RN*2 allele was associated with a decreased risk of Gram-negative BSI (unadjusted: HzR; 0.29, CI; 0.10–0.85; *p*-value; 0.025, adjusted: HzR; 0.31, CI; 0.11–0.88; *p*-value; 0.029). The same analysis for the IL1RN*3 allele and Gram-positive BSI could not be performed since none of these children had any episodes of Gram-positive BSI.

## 4. Discussion

Treatment-related infections are still a major concern during ALL treatment as it negatively affects morbidity and mortality. Risk-stratification could improve management of FN in the immunosuppressed child and previous studies have indicated a role for *TLR4*, *MBL*, and *IL-IRa* sequence variants as risk factors for serious infections in immunocompromised children. Contrary to previous findings, the present study demonstrates that two specific sequence variants in the *TLR4* increased the risk of viral associated FN, whilst a sequence variant in the *IL-1Ra* gene decreased the risk of bacterial BSI. Sequence variants in *MBL* did not affect FN or any of the MDI.

Several previous studies have reported that viral (mainly respiratory) infections are the most commonly verified infection (44–50%) during FN [6,29,30,31,32,33]. The lower proportion of viral infections in this study could be explained by different methods used for viral detection during the study period and that viral sampling was less stringent compared to blood culture sampling. Bacterial BSI were reported in 16.1% of the FN in the present study, which is in concordance with other studies [29,30,34]. Notably, in the current study, only ~40% of the FN had a proven etiology, which is a limitation of our study. However, it also highlights the importance of improved methods for microbial detection in children with ALL as suggested by us previously [35].

Next, we investigated whether genetic sequence variants in the innate immune proteins alter the risk of FN and associated MDI and thus, could be implemented as a risk-stratification tool. A previous study reported that *TLR4* sequence variants (rs10759931, rs11536889, rs6478317, and rs1927911) increased the risk of neutropenia in children treated for ALL [13]. Herein, we show that both the rs10759931 and rs11536889 increase the risk of viral infections during FN, but not to the number of episodes of FN in general. Since earlier studies mainly have associated *TLR4* sequence variants to the increased risk of Gram-negative bacterial infections, these findings were rather surprising [11,12,36,37]. Albeit, Awomoyi et al. reported that *TLR4* sequence variants (rs4986790 and rs4986791) were associated with symptomatic RSV infection in otherwise healthy infants [38].

Sequence variants in the gene coding for *IL-1Ra* have been investigated mainly in adult cohorts. To our knowledge, only one study has investigated the *IL-1Ra* in children treated for ALL, showing that children heterozygous or homozygous for the IL1RN*2 had an increased risk of developing septic shock [7]. On the contrary, the present study identified a decreased risk of bacterial BSI in children with IL1RN*2 and IL1RN*3 alleles. However, the definition of bacterial BSI and septic shock is not identical, hampering the comparisons of these two studies. Previous studies investigating *MBL* deficiency in children with cancer have reported contradictory results [8,9,10,11,14,15,39], whilst we could not show any association between *MBL* sequence variants and FN during the treatment for childhood ALL. Consequently, the present study strengthens the evidence that *MBL* does not play a role during episodes of FN and associated MDI. 

Despite our findings, one outstanding issue when investigating the role of gene sequence variants is the limited knowledge of how these findings correspond to phenotype and protein levels in vivo [16,40,41]. For *IL-1Ra*, it is not yet known whether high or low levels of the protein would increase or decrease the risk of bacterial BSI and sepsis. The development of sepsis occurs due to the pathogens itself and to an excessive inflammatory response by the immune system. Therefore, are high or low levels of *IL-1Ra* favorable or not in terms of sepsis development? Unfortunately, this could not be addressed by us as no plasma proteins were measured simultaneously. Another possible confounder is the redundancy in the immune system where previous studies have shown that the *IL-1Ra* gene not only affects the production of *IL-1Ra*, but also the *IL-1α* levels, and that the gene coding for *IL-1β* also may affect the *IL-1Ra* production [40,41]. The clinical utility of assessing gene variants in innate immune molecules is also hampered by many different outcome measurements in studies concerning children with ALL. When searching the literature to identify additional gene variants to include in this study, we identified two studies, investigating *TLR9* and *lymphotoxin-alpha* respectively, but with induction mortality as primary focus [42,43]. This was not possible in our cohort since we lacked induction deaths.

To summarize, the investigated gene sequence variants did not associate to increased risk for repeated episodes of FN, nor to increased risk for bacterial etiology as to why the clinical utility as a risk stratification tool is low. Most episodes of FN were however classified as fever of unknown origin which also may affect the clinical usefulness of these sequence variants for risk stratification. Our study also shows the need for stringent sampling and improved microbial detection methods in immunosuppressed children with FN to properly assess causality between etiology and fever.

## Figures and Tables

**Table 1:** Patient characteristics of the 122 included children treated for ALL.

Characteristics	*n* (%)
Gender	
Female	58 (47.5)
Male	64 (52.4)
Risk Group	
Standard risk	54 (44.3)
Intermediate risk	48 (39.3)
High risk	20 (16.4)
Protocol	
NOPHO ALL-2000	43 (35.2)
NOPHO ALL-2008	79 (64.8)
Phenotype of ALL	
Pre-B ALL	106 (86.9)
T-cell ALL	16 (13.1)
Outcome	
Relapse *	8 (6.6)
HSCT	2 (1.6)
Death during follow up	2 (1.6)
	Median (range; IQR)
Age at diagnosis (years)	5.4 (1.1–17.9; 3.3–10.4)

* within 2.5 years from diagnosis. Abbreviations: ALL, Acute lymphoblastic leukemia; HSCT, Hematopoietic stem-cell transplantation; IQR, interquartile range.

**Table 2:** Association between sequence variants in genes coding for *TLR4*, *MBL*, and *IL-1Ra* and the risk of FN and specific MDI during pediatric ALL treatment.

	Febrile Neutropenia (*n* = 380)		Bacterial BSI (*n* = 61)		Viral Infection (*n* = 64)	
Sequence Variant	Unadjusted	Adjusted	Unadjusted	Adjusted	Unadjusted	Adjusted
*TLR4* rs2737190	1.03 (0.78–1.35; 0.83)	1.01 (0.82–1.24; 0.93)	1.02 (0.59- 1.75; 0.94)	0.93 (0.59–1.46; 0.74)	0.88 (0.48–1.62; 0.69)	0.96 (0.56–1.66; 0.89)
*TLR4* rs1927911	0.93 (0.71–1.21; 0.6)	0.96 (0.78–1.18; 0.69)	0.98 (0.57–1.70; 0.95)	0.97 (0.61–1.55; 0.90)	0.70 (0.39–1.27; 0.24)	0.74 (0.43–1.26; 0.27)
*TLR4* rs10759931	0.93 (0.71–1.23; 0.62)	0.94 (0.77–1.15; 0.55)	0.89 (0.50–1.61; 0.71)	0.88 (0.56–1.41; 0.60)	**2.46 (1.17–5.18; 0.017)**	**2.38 (1.24–4.57; 0.0094)**
*TLR4* rs11536889	0.96 (0.72–1.28; 0.79)	1.09 (0.86–1.39; 0.45)	0.82 (0.46–1.45; 0.49)	1.02 (0.59–1.76; 0.94)	1.59 (0.86–2.96; 0.14)	**1.94 (1.14–3.31; 0.015)**
*TLR4* rs4986790	1.04 (0.63–1.72; 0.87)	1.04 (0.73–1.48; 0.84)	1.25 (0.52–2.99; 0.62)	1.23 (0.63–2.39; 0.54)	0.80 (0.28–2.27; 0.68)	0.92 (0.35–2.39; 0.87)
*TLR4* rs4986791	0.94 (0.61–1.45; 0.79)	0.90 (0.66–1.22; 0.48)	0.93 (0.42–2.04; 0.85)	0.80 (0.44–1.47; 0.48)	0.73 (0.29–1.85; 0.5)	0.74 (0.31–1.75; 0.49)
*TLR4* rs4986790 and rs4986791	0.96 (0.64–1.45; 0.86)	0.94 (0.70–1.27; 0.68)	1.18 (0.57–2.45; 0.66)	1.06 (0.58–1.91; 0.85)	0.69 (0.27–1.76; 0.43)	0.71 (0.30–1.70; 0.44)
*MBL* rs1800450, rs5030737 and rs1800451	1.09 (0.83–1.43; 0.53)	1.09 (0.89–1.35; 0.41)	0.70 (0.37–1.32; 0.27)	0.78 (0.44–1.38; 0.39)	0.78 (0.37–1.67; 0.53)	0.79 (0.40–1.58; 0.51)
IL1RN*2	1.08 (0.83–1.41; 0.55)	1.07 (0.86–1.34; 0.52)	**0.49 (0.28–0.88; 0.016)**	**0.51 (0.31–0.84; 0.009)**	1.65 (0.88–3.10; 0.12)	1.47 (0.84–2.58; 0.17)
IL1RN*3	1.24 (0.58–2.68; 0.58)	0.87 (0.58–1.30; 0.49)	0.25 (0.04–1.54; 0.14)	**0.16 (0.05–0.51; 0.0022)**	1.72 (0.62–4.74; 0.3)	1.09 (0.47–2.54; 0.85)

Results are presented as HzR (CI; and *p-*value); Adjusted; for age, protocol, and risk group for FN and bacterial BSI and only for age and protocol for viral infection. All infectious episodes were analyzed as only bacterial BSI (viral and IFD excluded) and only viral infections (bacterial and IFD excluded). Significant gene sequence variants for the respective MDI in bold.

## Supplementary Materials

The following are available online at https://www.mdpi.com/2227-9067/7/12/296/s1, Table S1: Pyrosequencing PCR-primers and primers for detection of sequence variants in the genes coding for *TLR4, MBL* and *IL-1Ra* (intron 2). Table S2: Sequencing primers and settings for pyrosequencing reactions. Table S3: Genotyping results *MBL* and *TLR4.* Table S4: Genotyping results *IL-1Ra* gene (IL1RN).
