# Supplementary material for: Genetic Sequence Variants in TLR4, MBL or IL-1 Receptor Antagonist is not Associated to Increased Risk for Febrile Neutropenia in Children with ALL
Source: Children (Basel). 2020 Dec 16;7(12):296. doi: 10.3390/children7120296 (PMC7766164; doi:10.3390/children7120296)
Supplement: Supplementary file 1 [file children-07-00296-s001.pdf]

Table S1. Pyrosequencing PCR-primers and primers for detection of sequence variants in the genes coding for *TLR4*, *MBL* and *IL-1Ra* (intron 2).

Gene	Sequence Variant	Primer Forward (5′–3′)	Primer Reverse (5′–3′)	Fragment Size
TLR4	rs2737190	[Bio]CCTGTGATGATTAGGGCTGAA	TCTGAACCACCTCTTCTACCTG	
TLR4	rs1927911	GGCAGTCAAGATGTCCAGACCT	[Bio]TGGGAATCCATGCACTCTAAA	
TLR4	rs10759931	GCAGGGTAAGCAGGGATAGGAC	[Bio]GCTTTTACACCCAAGTAGACACCG	
TLR4	rs11536889	[Bio]GGGCAATGCTCCTTGACCA	TTTCCCTGATGACATCCTGATTG	
MBL	rs1800450	[Bio]GACCTGCCCTGCAGTGATTG	CGTACCTGGTTCCCCCTTTT	
MBL	rs1800737	CAGTGATTGCCTGTAGCTCTCCA	[Bio]GAGACAGAACAGCCCAACACG	
MBL	rs1800451	AAGATGGGCGTGATGACACC	[Bio]GCCCAACACGTACCTGGTTC	
IL-1Ra		CTCAGCAACACTCCTAT	TCCTGGTCTGCAGGTAA	240 bp, 410 bp, 500 bp

Abbreviations; [Bio]Biotinylated primer, Bp, Base pair, *IL1-Ra*; *IL-1* receptor antagonist, *MBL*; Mannose binding lectin, *TLR4*; Toll-like receptor 4.

Table S2. Sequencing primers and settings for pyrosequencing reactions.

Target Sequence Variant	Sequencing Primer (5′-3′)	Sequence to Analyze	Dispensation Order
rs2737190	GCTTTTACACCCAAGTAGAC	ACC/TGTTATT	GACTCGTAT
rs1927911	TGACAACTGCATTCTTTT	C/TCTTGGCTC	GTCATGCTC
rs10759931	AGGGTCTGTCTCTAGTTGT	CTGA/GTACC	GCTGAGTAC
rs11536889	TCTCAATGATAACATCCACT	C/GTTCCCAAATG	ACGATCATG
rs1800450	CCTTTTCTCCCTTGG	TGT/CCATCACGCCC	CTGTCGATC
rs1800737	TTCCCAGGCAAAGAT	GGGT/CGTGA	AGTCAGTGA
rs1800451	GCGTGATGACACCAAG	GG/AAGAAAAGGGGAACC	CGACGAAGAC

Table S3. : Genotyping results *MBL* and *TLR4*.

Sequence variant	Major Allele	Minor Allele	Major Allele Frequency	Minor Allele Frequency	Wild Type (*n*)	Heterozygous (*n*)	Homozygous (*n*)
TLR4 rs2737190	T	C	0.66	0.34	54	52	16
TLR4 rs1927911	C	T	0.68	0.32	57	51	14
TLR4 rs10759931	G	A	0.59	0.41	45	54	23
TLR4 rs11536889	C	G	0.83	0.17	81	40	1
TLR4 rs4986790	A	G	0.96	0.04	111	10	0
TLR4 rs4986791	C	T	0.95	0.05	109	13	0
MBL rs1800450	C	T	0.84	0.16	86	34	2
MBL rs5030737	C	T	0.98	0.02	117	5	0
MBL rs1800451	G	A	0.99	0.01	120	1	1

9 children had both *TLR4* rs4986790 and *TLR4* rs4986791, 3 children had both *MBL* rs1800450 and *MBL* rs5030737 and one had both *MBL* rs5030737 and *MBL* rs1800451 (homozygous). Additionally, children could harbor > 1 of the sequence variants in the *TLR4* gene (data not shown) and also > 1 of the different sequence variants in *TLR4*, *MBL* and *IL1RN* (data not shown). Abbreviations: *n*; number of individuals, *MBL*; Mannose binding lectin, *TLR4*; Toll like receptor 4.

Table S4. Genotyping results *IL-1Ra* gene (*IL1RN*).

IL1RN allele	Frequency *IL1RN*1*	Frequency *IL1RN**2 or *3	Wild type (*n*)	Heterozygous (*n*)	Homozygous (*n*)
*IL1RN**2	0.75	0.25	72	38	11
*IL1RN**3	0.97	0.03	114	7	0

One child had both one *IL1RN**2 and *IL1RN*3*. Children could harbor > 1 of the different sequence variants in *TLR4*, *MBL* and *IL1RN* (data not shown). Abbreviations: *IL1RN*; *IL-1* Receptor antagonist gene, *n*; number of individuals.
